# Bedside ultrasound to detect central venous catheter misplacement and associated iatrogenic complications: a systematic review and meta-analysis

**DOI:** 10.1186/s13054-018-1989-x

**Published:** 2018-03-13

**Authors:** Jasper M. Smit, Reinder Raadsen, Michiel J. Blans, Manfred Petjak, Peter M. Van de Ven, Pieter R. Tuinman

**Affiliations:** 1 Department of Intensive Care Medicine, Research VUmc Intensive Care (REVIVE), VU University Medical Center, De Boelelaan 1117, 1081 HV Amsterdam, The Netherlands; 2 Institute for Cardiovascular Research (ICAR-VU), VU University Medical Center, De Boelelaan 1117, 1081 HV Amsterdam, The Netherlands; 3 Department of Intensive Care Medicine, Rijnstate Hospital, Wagnerlaan 55, 6815 AD Arnhem, The Netherlands; 4 Department of Intensive Care medicine, Groene Hart Ziekenhuis, Bleulandweg 10, 2803 HH Gouda, The Netherlands; 5 Department of Epidemiology and Biostatistics, VU University Medical Center, De Boelelaan 1117, 1081 HV Amsterdam, The Netherlands

**Keywords:** Central venous catheter, Ultrasound, CVC malposition, Iatrogenic complications, Chest x-ray, Pneumothorax, Meta-analysis

## Abstract

**Background:**

Insertion of a central venous catheter (CVC) is common practice in critical care medicine. Complications arising from CVC placement are mostly due to a pneumothorax or malposition. Correct position is currently confirmed by chest x-ray, while ultrasonography might be a more suitable option. We performed a meta-analysis of the available studies with the primary aim of synthesizing information regarding detection of CVC-related complications and misplacement using ultrasound (US).

**Methods:**

This is a systematic review and meta-analysis registered at PROSPERO (CRD42016050698). PubMed, EMBASE, the Cochrane Database of Systematic Reviews, and the Cochrane Central Register of Controlled Trials were searched. Articles which reported the diagnostic accuracy of US in detecting the position of CVCs and the mechanical complications associated with insertion were included. Primary outcomes were specificity and sensitivity of US. Secondary outcomes included prevalence of malposition and pneumothorax, feasibility of US examination, and time to perform and interpret both US and chest x-ray. A qualitative assessment was performed using the QUADAS-2 tool.

**Results:**

We included 25 studies with a total of 2548 patients and 2602 CVC placements. Analysis yielded a pooled specificity of 98.9 (95% confidence interval (CI): 97.8–99.5) and sensitivity of 68.2 (95% CI: 54.4–79.4). US examination was feasible in 96.8% of the cases. The prevalence of CVC malposition and pneumothorax was 6.8% and 1.1%, respectively. The mean time for US performance was 2.83 min (95% CI: 2.77–2.89 min) min, while chest x-ray performance took 34.7 min (95% CI: 32.6–36.7 min). US was feasible in 97%. Further analyses were performed by defining subgroups based on the different utilized US protocols and on intra-atrial and extra-atrial misplacement. Vascular US combined with transthoracic echocardiography was most accurate.

**Conclusions:**

US is an accurate and feasible diagnostic modality to detect CVC malposition and iatrogenic pneumothorax. Advantages of US over chest x-ray are that it can be performed faster and does not subject patients to radiation. Vascular US combined with transthoracic echocardiography is advised. However, the results need to be interpreted with caution since included studies were often underpowered and had methodological limitations. A large multicenter study investigating optimal US protocol, among other things, is needed.

**Electronic supplementary material:**

The online version of this article (10.1186/s13054-018-1989-x) contains supplementary material, which is available to authorized users.

## Background

Most patients admitted to an intensive care unit (ICU) undergo central venous catheterization. In the United States, over 5 million central venous catheter (CVC) placements are performed each year [1]. Although central venous catheterization offers multiple advantages, the procedure is associated with adverse events that could be hazardous for patients. Adverse events can be divided into immediate complications and delayed complications. Immediate complications arise directly after introducing a CVC and consist of mechanical complications and malposition. The most common mechanical complications include arterial puncture, hematoma, and pneumothorax [2, 3]. Delayed complications consist of infectious and thrombotic adverse events and may be provoked by malposition of a CVC [4]. Additionally, malposition of the CVC tip into the right atrium could cause arrhythmias and atrial perforation [5].

To date, the most commonly used reference standard to detect CVC malposition and pneumothorax is post-procedural chest x-ray (CXR). A disadvantage of CXR, however, is that the patient is exposed to radiation. Moreover, performing and interpreting the CXR are often time consuming. Replacing or omitting CXR could reduce healthcare costs and minimize the delay until catheter use [6].

To confirm correct intravascular catheter position and to detect pneumothorax it has been suggested that ultrasound (US) may be a suitable alternative diagnostic modality. Major advantages of US over CXR are that it is often performed faster and does not subject a patient to radiation [7]. Furthermore, using US to guide cannulation is considered as best practice nowadays and, compared with the traditional ‘blind’ landmark method, it reduces failed catheterizations and complications [8, 9]. To verify correct CVC placement, the accuracy of bedside US as an alternative diagnostic modality has been analyzed by a number of small studies. However, these studies used different US protocols and reported a wide range of diagnostic accuracy. To address this problem we performed a systematic review and meta-analysis on these studies.

The primary aim of our study was to investigate whether intravascular CVC misplacement and pneumothorax can be reliably detected by US. A secondary aim was to compare the diagnostic outcomes of the studies to their respective US protocol. Outcomes were compared to a reference standard, e.g., CXR or transesophageal echocardiography (TEE).

## Methods

### Study design

This is a systematic review and meta-analysis.

To improve the quality of this systematic review we followed the PRISMA guidelines (Preferred Reporting Items for Systematic Reviews and Meta-Analyses) [10] (Additional file 1). The protocol was registered at PROSPERO International prospective register of systematic reviews, registration number CRD42016050698.

### Selection of studies

We aimed to include all studies that investigated the accuracy of bedside US in detecting CVC misplacement and other iatrogenic complications, e.g., pneumothorax. In these studies, US is compared to any diagnostic modality that detects CVC malposition. To select eligible studies, a medical librarian who is experienced in organizing systematic reviews was consulted to define and perform a robust search strategy. The search was implemented in MEDLINE via PubMed, EMBASE, the Cochrane Database of Systematic Reviews, and the Cochrane Central Register of Controlled Trials. The initial search was performed on 4 October 2016 and a second search was performed on 9 January 2017 (Additional file 2).

### Inclusion of studies

Titles and abstracts were evaluated by one independent reviewer (JMS) while a full-text analysis was performed by two independent reviewers (JMS and RR). References of the selected studies were screened and potentially eligible studies were evaluated and included or excluded afterwards. Inclusion and exclusion criteria are described in Additional file 3. For the removal of duplicates, Covidence® (systematic review software) was used. Disagreement was resolved by consensus meetings with a third reviewer (PRT).

### Data extraction

Data were extracted independently by two reviewers (JMS and RR) using Covidence®. The following study characteristics from the included studies were collected: first author and year of publication, study design and period, setting and country, number of patients and CVCs, utilized US protocol, reference standard, primary outcome and secondary outcome of individual studies, number of performing operators, and number of experienced operators. To be classified as experienced, US operators were required to have completed an IC US course and at least 20 practice studies [11]. In addition, characteristics of patients and outcome parameters were collected, including gender, age, weight/body mass index (BMI) and CVC insertion site. These characteristics are described in Additional file 4. To calculate specificity and sensitivity, a 2 × 2 contingency table was constructed based on the raw data from the included literature. Raw data comprise the number of true positives, true negatives, false positives, and false negatives of the included studies concerning US. If additional information was required, attempts were made to contact the authors of the article.

### Outcomes

Our primary outcome was to evaluate the accuracy of bedside US in detecting CVC misplacement. If an alternative primary outcome rather than CVC malposition, for example CVC tip visualization or inter-observer agreement, was reported by the included studies, raw data were adjusted or reversed to meet our outcome specifications. The specificity and sensitivity of the included studies were calculated by extracting the raw data and implementing it in a 2 × 2 contingency table. A ‘true positive’ result was defined as a US-suggested aberrant position of the CVC (catheter tip in any other vein than the superior vena cava, outside the venous system, or positioned in the right atrium), confirmed by a reference test. If bedside US correctly ruled out an aberrant position of the catheter tip, as confirmed by a reference standard, it was considered to be a ‘true negative’ result.

The feasibility of US was defined as the percentage of patients in whom cardiac US images could be obtained during transthoracic echocardiography (TTE). Both feasibility and accuracy of lung US to detect pneumothorax were regarded as secondary outcome measures. Since CXR may be an inadequate reference standard, we could not accurately determine accuracy parameters of lung US to identify pneumothorax [12]. Moreover, to detect pneumothorax a recent meta-analysis showed a better sensitivity and a similar specificity for lung US in comparison to CXR [13]. Therefore, the prevalence of pneumothorax was reported instead. Finally, the time to perform the US examination and time to perform or interpret CXR (whichever was reported) were regarded as a secondary outcome measure.

US protocols of included studies could be divided into four separate US protocols, consisting of 1) vascular US and TTE; 2) TTE combined with contrast-enhanced US (CEUS); 3) a combination of 1 and 2; or 4) supraclavicular US (SCU).

CEUS is defined as a flush of the CVC with agitated or non-agitated saline during TTE. A subgroup analysis was conducted on these four protocols. In addition, it was noted whether the US examination was performed during central venous cannulation and therefore the advancement of the guidewire was primarily visualized, or whether the examination was performed post-procedural and CEUS was utilized to identify catheter tip position. Finally, accuracy of US to detect intra-atrial CVC misplacement was investigated. To perform this analysis, a distinction was made between intra- and extra-atrial misplacement [14–16]. Extra-atrial misplacements were considered to be all vascular misplacements other than intra-cardiac position of the CVC tip. An additional subgroup analysis was conducted in these two groups.

### Quality assessment

The QUADAS-2 tool (Quality Assessment of Diagnostic Accuracy Studies) was utilized by two independent reviewers (JMS and RR) for quality assessment of the included studies. Disagreement was resolved by consensus meetings with a third reviewer (PRT). Additional file 5 contains a complete overview of the quality assessment.

### Statistical analysis and data synthesis

Specificity and sensitivity were first estimated separately for each study, together with their 95% exact confidence interval (CI). In the event that specificity and sensitivity were estimated as 100%, a one-sided 97.5% exact CI was calculated instead. These analyses were performed in Stata 14. A pooled estimate for specificity and a 95% CI was obtained using separate generalized estimating equations (GEEs) on individual patient data assuming an exchangeable correlation structure for outcomes of patients included in the same study. The same procedure was used for sensitivity. Forest plots were made using the calculated 95% CIs. GEE analyses were performed and plots were made in SPSS 22. Publication bias was assessed using Deek’s test. The secondary outcomes of mean time of US performance, mean time to CXR performance, and mean time to CXR interpretation were summarized by weighted means, together with their 95% CIs where weights were set equal to the inverse of the standard error of the mean reported in the study.

## Results

**Fig. 1:**
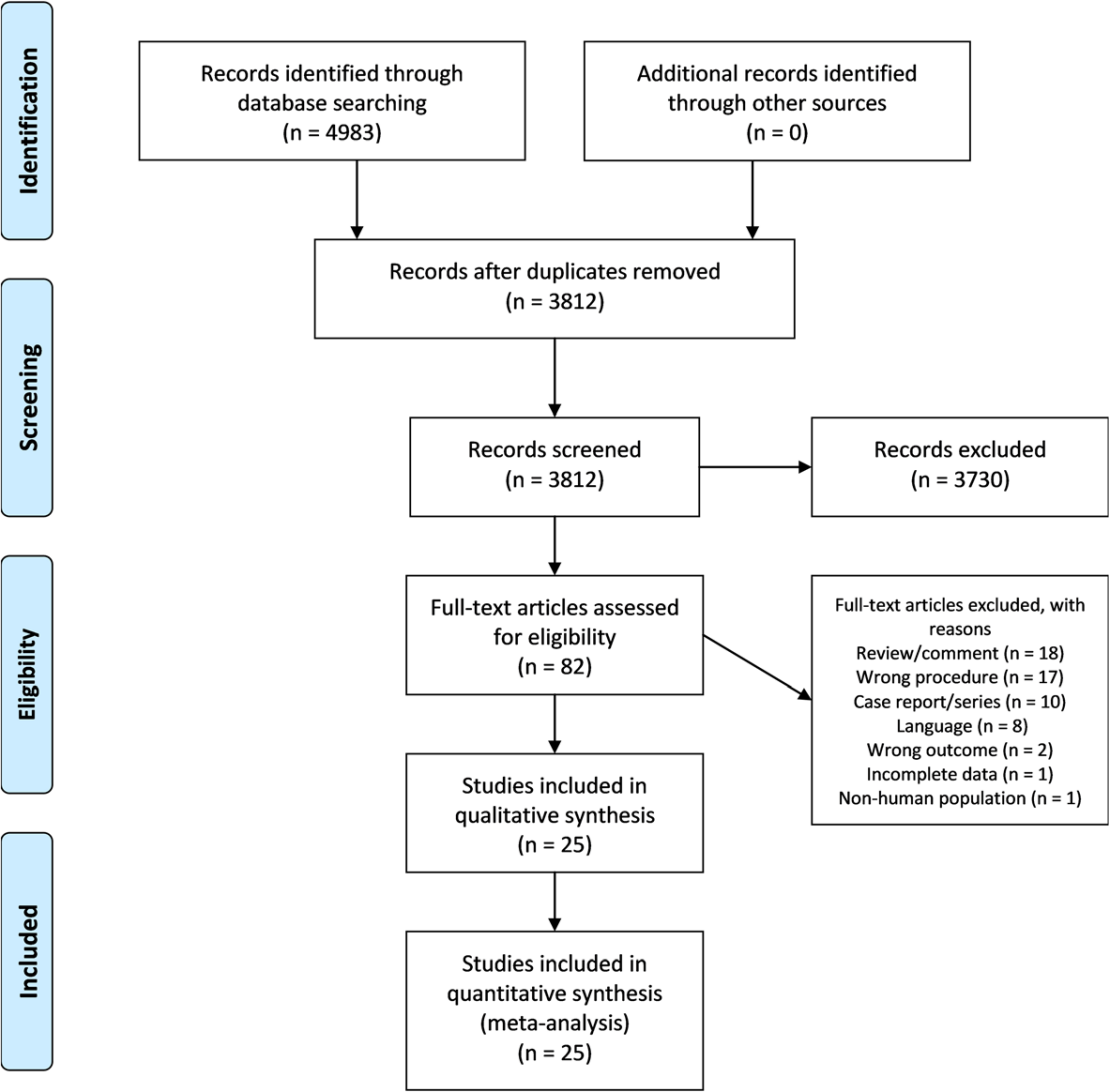
PRISMA flow diagram of search strategy and study selection. Depicted in the flow diagram are the number of identified records, the number of screened records, the number of articles assessed for eligibility with reasons for exclusion, and the number of studies included in the qualitative and quantitative syntheses

Details regarding search and study selection are presented in Fig. 1. Of the initial 4983 articles identified, 25 articles, with a total of 2548 patients and 2602 CVC placements, met the inclusion criteria.

### Study characteristics

**Fig. 2:**
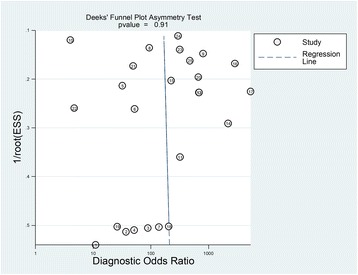
Deek’s funnel plot asymmetry test for all 25 studies. The risk of bias when all 25 studies are included in Deek’s funnel plot asymmetry test (*p* = 0.91). ESS effective sample size

**Fig. 3:**
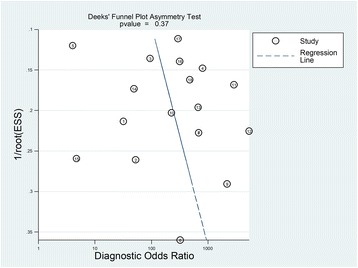
Deek’s funnel plot asymmetry test for 18 studies in which both specificity and sensitivity could be estimated. The risk of bias when only the 18 studies are included in Deek’s funnel plot asymmetry test for which both sensitivity and specificity could be estimated (*p* = 0.37). ESS effective sample size

Study characteristics are shown in Table 1. CVC position was evaluated prospectively in 21 studies; furthermore, there were three pilot studies and one retrospective study. The majority of studies used CXR as a reference test to evaluate CVC position; additionally, TEE was used in two studies, additional intra-fluoroscopy in one, and TEE was used exclusively in one study. US protocols used were a combination of vascular US and TTE (*n* = 6), a combination of TTE and CEUS (*n* = 11), a combination of vascular US, TTE, and CEUS (*n* = 5), and a SCU approach in the remaining studies (*n* = 3). In four studies advancement of the guidewire was assessed by US, whereas in the remaining 21 studies the CVC was visualized by US directly after placement. In one study the accuracy of CXR to detect CVC malposition was investigated and US was used as a reference standard. Here, we reversed the outcome and we used the raw data in our meta-analysis [17]. Most studies had less than three operators. Exact operator experience was stated in 19 studies. Patient characteristics are shown in Additional file 4. Deek’s test did not show evidence of strong publication bias (*p* = 0.91 for all 25 studies and *p* = 0.37 for 18 studies in which both specificity and sensitivity could be estimated; Fig. 2 and Fig. 3).

**Table 1:** Study characteristics

Author (year)	Study design (period)^1^	Setting (country)	Patients (CVCs), *n*	Ultrasoundprotocol	Reference standard	Primary outcome (secondary outcome)	Performing operators (experienced operators), *n*
Killu et al. (2010) [47]	Prospective pilot study	Surgical ICU (United States)	5 (5)	Supraclavicular ultrasound^a^	CXR	Malposition (time advancement guidewire)	1 (1)
Kim et al. (2015) [26]	Prospective pilot study (Jul 2012–Oct 2012)	Operating theatres (Germany)	51 (51)	Supraclavicular ultrasound^b^	CXR/TEE	Malposition (time until confirmation)	2 (2)
Kim et al. (2016) [25]	Prospective pilot study(Jun 2014–Aug 2014)	Operating theatres (Germany)	20 (20)	Supraclavicular ultrasound^b^	CXR	Malposition (time advancement guidewire)	1 (1)
Baviskar et al. (2015) [48]	Prospective study (Apr 2013–Jan 2014)	Surgical ICU (India)	25 (25)	TTE and CEUS^a^	CXR	Time until confirmation (malposition)	1 + (1+)^3^ experienced staff
Cortellaro et al. (2014) [49]	Prospective study	Emergency department (Italy)	71 (71)	TTE and CEUS^a^	CXR	Malposition (time until confirmation)	3 (2)
Duran-Gehring et al. (2015) [50]	Prospective study (Dec 2012–Nov 2013)	Emergency department (United States)	50 (50)	TTE and CEUS^a^	CXR	Time until confirmation (malposition, pneumothorax)	2 (2)
Gekle et al. (2015) [51]	Prospective study (Dec 2012–Mar 2014)	Emergency department (United States)	81 (81)	TTE and CEUS^a^	CXR	Malposition, pneumothorax (time until confirmation)	Unclear
Kamalipour et al. (2016) [52]	Prospective study (Aug 2013–Jan 2014)	Operating theatres (Iran)	116 (116)	TTE and CEUS^a^	CXR	Malposition	1 (1)
Lanza et al. (2006) [53]	Prospective study (Nov 2004–Sep 2005)	Pediatric ICU (Italy)	107 (107)	TTE and CEUS^a^	CXR	Malposition, pneumothorax	1 (1)
Salimi et al.^2^ (2015) [17]	Prospective study	Nephrology department (Iran)	82 (82)	TTE and CEUS^a^	CXR	Malposition, pneumothorax	1 (1)
Santarsia et al. (2000) [54]	Prospective study	Nephrology department (Italy)	158 (158)	TTE and CEUS^a^	CXR	Malposition	Unclear
Weekes et al. (2014) [55]	Prospective study (Jan 2013–Apr 2013)	Emergency department and ICU	147 (152)	TTE and CEUS^a^	CXR	Malposition	5 (5)
Weekes et al. (2016) [56]	Prospective study (Nov 2013–Mar 2015)	Emergency department and ICU	156 (156)	TTE and CEUS^a^	CXR	Malposition, pneumothorax (time until confirmation)	Unclear, by or under supervision of study investigator
Wen et al. (2014) [57]	Retrospective study (Jun 2011–Jul 2012)	Nephrology department (Germany)	202 (219)	TTE and CEUS^a^	CXR	Malposition (time until confirmation)	2 + (2+)^3^
Alonso-Quintela et al. (2015) [58]	Prospective study (Jan 2012–Jan 2014)	Pediatric ICU (Spain)	40 (51)	Vascular ultrasound and TTE^a^	CXR	Malposition (time until confirmation)	1 (1)
Maury et al. (2001) [59]	Prospective study (Mar 1999–Sep 1999)	ICU (France)	81 (85)	Vascular ultrasound and TTE^a^	CXR	Malposition, pneumothorax (time until confirmation)	3 (0)
Miccini et al. (2016) [46]	Prospective study (Jan 2012–Dec 2014)	Operating theatres (Italy)	302 (302)	Vascular ultrasound and TTE^a^	IF/CXR	Malposition, pneumothorax	2 (2)
Park et al. (2014) [60]	Prospective study	Pediatric ICU (United States)	108 (108)	Vascular ultrasound and TTE^a^	CXR	Malposition (insertion depth CVC)	3 (3)
Arellano et al. (2014) [27]	Prospective study	Operating theatres (Canada)	100 (100)	Vascular ultrasound and TTE^b^	TEE	Malposition	4 (2)
Bedel et al. (2013) [24]	Prospective study (Jan 2010–Nov 2010)	ICU (France)	98 (101)	Vascular ultrasound and TTE^b^	CXR	Malposition (pneumothorax, time until confirmation)	1 (1)
Blans et al. (2016) [18]	Prospective study (Jan 2015–Sep 2015)	ICU (The Netherlands)	53 (53)	Vascular ultrasound, TTE and CEUS^a^	CXR	Malposition, pneumothorax (time until confirmation)	2 (2)
Matsushima and Frankel (2010) [19]	Prospective study (Nov 2004–Sep 2005)	Surgical ICU (United States)	69 (83)	Vascular ultrasound, TTE and CEUS^a^	CXR	Malposition, pneumothorax (time until confirmation)	1 (0)
Meggiolaro et al. (2015) [32]	Prospective study (Jan 2013–Sep 2013)	Operating theatres (Italy)	105 (105)	Vascular ultrasound, TTE and CEUS^a^	CXR	Malposition, pneumothorax (timing bubble test, time until confirmation)	1 (1)
Vezzani et al. (2010) [31]	Prospective study (Apr 2008–Aug 2008)	ICU (Italy)	111 (111)	Vascular ultrasound, TTE and CEUS^a^	CXR	Malposition, pneumothorax (time until confirmation, cost analysis)	1 (1)
Zanobetti et al. (2013) [61]	Prospective study (Jan 2009–Dec 2011)	Emergency department (Italy)	210 (210)	Vascular ultrasound, TTE and CEUS^a^	CXR	Malposition, pneumothorax (time until confirmation)	4 + (4+)^3^

*CEUS* contrast enhanced ultrasound, *CVC* central venous catheter, *CXR* chest x-ray, *ICU* intensive care unit, *IF* intra-fluoroscopy, *TEE* transesophageal echocardiography, *TTE* transthoracic echocardiography

^a^The CVC is primarily visualized

^b^The advancement of the guidewire is primarily visualized

^1^All studies were observational in design

^2^Accuracy CXR investigated; TTE used as reference standard

^3^Possible more than described amount of operators

### Outcomes

**Fig. 4:**
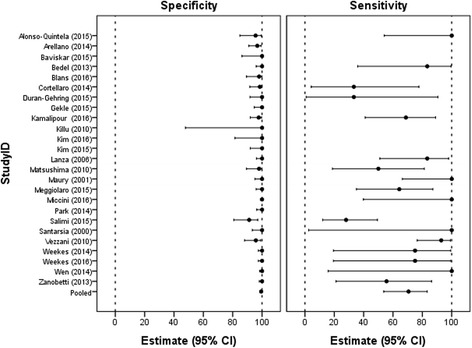
Forest plot of the specificity and sensitivity of ultrasound for detection of CVC-related complications. The pooled specificity and sensitivity as well as the specificity and sensitivity for each study individually with their respective confidence interval (CI). Studies showed significant statistical heterogeneity; for specificity, *I*^2^ = 83.3 (95% CI: 64.6–86.7) and, for sensitivity, *I*^2^ = 75.5 (95% CI: 77.1–90.4)

The results of primary and secondary outcomes of included studies are presented in Table 2. Pooled specificity was 98.9 (95% CI: 97.8–99.5), and the lowest specificity reported was 91.2 (95% CI: 80.7–97.1) by Salimi et al. [17]. Pooled sensitivity was 68.2 (95% CI: 54.4–79.4), and the lowest sensitivity reported was 0 (95% CI: 0–70.8) by Blans et al. [18]. Pooled specificity and sensitivity of US for detection of CVC misplacement are shown in a Forest plot in Fig. 4. Specificity and sensitivity show considerable statistical heterogeneity: for specificity, *I*^2^ = 83.3 (95% CI: 64.6–86.7) and, for sensitivity, *I*^2^ = 75.5 (95% CI: 77.1–90.4). On average, US examination was feasible in 96.8% of the cases. The lowest reported feasibility of 71% was reported by Matsushima and Frankel [19]. The prevalence of pneumothorax, investigated by 11 studies, was 1.1% on average. In addition, the prevalence of CVC malposition was 6.8% on average.

**Table 2:** Outcomes regarding feasibility, prevalence, accuracy parameters, and time to measurement of included studies

Study	Feasibility	Prevalence of pneumothorax (%)	Prevalence of malposition (%)	Specificity (95% CI)^1^	Sensitivity (95% CI)^2^	Mean time for US (min) (±SD)^4^ [IQR]	Mean time for CXR performance (min) (±SD)^4^ [IQR]	Mean time for CXR interpretation (min) (±SD)^4^ [IQR]
Killu et al. (2010) [47]	100%	–	0%	100.0 (47.8–100)	–	4.2	–	–
Kim et al. (2015) [26]	92%	–	0%	100 (92.0–100)	–	11 (0.72)	111 (31)	–
Kim et al. (2016) [25]	100%	–	0%	100 (81.5–100)	–	–	–	–
Baviskar et al. (2015) [48]	100%	–	0%	100 (86.3–100)	–	0.75 (0.25)	–	–
Cortellaro et al. (2014) [49]	100%	–	8.4%	98.5 (91.7–100)	33.3 (4.3–77.7)	4 (1)	–	288 (216)
Duran-Gehring et al. (2015) [50]	92%	4.3%	6.5%	100 (91.8–100)	33.3 (0.8–90.6)	5 (0.8)	28.2 (11.3)	299 (90.5)
Gekle et al. (2015) [51]	100%	0%	0%	100 (94.7–100)	–	8.80 (1.34)		45.78 (8.75)
Kamalipour et al. (2016) [52]	89.7%	–	15.4%	97.7 (92.0–99.7)	68.8 (41.0–89.0)	–	–	–
Lanza et al. (2006) [53]	100%	0.9%	11.2%	100 (96.2–100)	83.3 (51.0–97.7)	–	–	–
Salimi et al.^*^ (2015) [17]	100%	–	30.5%	91.2 (80.7–97.1)	28.0 (12.1–49.4)	–	–	–
Santarsia et al. (2000) [54]	100%	–	1.9%	100 (93.3–100)	100 (2.5–100)	–	–	–
Weekes et al. (2014) [55]	96.6%	–	2.7%	100 (97.5–100)	75.0 (19.4–99.4)	–	–	–
Weekes et al. (2016) [56]	97.4%	–	2.6%	100 (97.5–100)	75.0 (19.4–99.4)	1.1 (0.7)	20 (30)	–
Wen et al. (2014) [57]	100%	–	0.9%	100 (98.3–100)	100 (15.8–100)	3.2 (1.1)	28.3 (25.7)	–
Alonso-Quintela et al. (2015) [58]	100%	–	11.8%	95.6 (84.9–99.5)	100 (54.1–100)	2.23 (1.06)	–	22.96 (20.43)
Maury et al. (2001) [59]	98.8%	1.2%	10.7%	100 (95.2–100)	100 (66.4–100)	6.8 (3.5)	80.3 (66.7)	–
Miccini et al. (2016) [46]	100%	1.0%	1.3%	100 (98.8–100)	100 (39.8–100)	–	–	–
Park et al. (2014) [60]	96.2%	–	0%	100 (96.4–100)	–	–	–	–
Arellano et al. (2014) [27]	94%	–	0%	96.8 (91.0–99.3)	–	–	–	–
Bedel et al. (2013) [24]	97%	0%	6.2%	100 (96.0–100)	83.3 (35.9–99.6)	1.76 (1.3)	49 (31)	103 (81)
Blans et al. (2016) [18]	98.1%	0%	5.8%	98.0 (89.4–99.9)	0 (0–70.8)	–	–	24.5 [18.1–45.3]
Matsushima and Frankel (2010) [19]	71%	0%	16.9%	98.0 (89.4–99.9)	50.0 (18.7–81.3)	10.8	–	75.3
Meggiolaro et al. (2015) [32]	100%	0%	13.3%	100 (96.0–100)	64.3 (35.1–87.2)	5.0 [5.0-10.0]	–	67.0 [42.0–84.0]
Vezzani et al. (2010) [31]	89.2%	1.8%	28.3%	95.8 (88.1–99.1)	92.9 (76.5–99.1)	10 (5)	83 (79)	–
Zanobetti et al. (2013) [61]	100%	2.0%	4.4%	100 (98.1–100)	55.6 (21.2–86.3)	5 (3)	–	65 (74)
Pooled (patients, *n*)		(patients, *n* = 1267)	(patients, *n* = 2548)			(patients, *n* = 1362)	(patients, *n* = 749)	(patients, *n* = 777)
All studies (2548)	96.8%	1.1%	6.8%	98.4 (97.8–99.5)	68.2 (54.4–79.4)	2.83 (95% CI: 2.77–2.89)	34.7 (95% CI: 32.6–36.7)	46.3 (95% CI: 44.4–48.2)
Supraclavicular ultrasound (76)	94.6%	–	0%	100 (94.4–100)^3^	–			
TTE and CEUS (1195)	97.7%	1.4%	6.8%	98.9 (96.1–99.7)	68.7 (61.7–96.4)			
Vascular ultrasound and TTE (729)	98.1%	0.8%	3.4%	99.0 (96.5–99.7)	96.1 (79.7–99.4)			
Vascular ultrasound, TTE and CEUS (548)	93.3%	1.4%	12.3%	98.6 (96.1–99.5)	56.2 (32.8–77.1)			

*CEUS* contrast enhance ultrasound, *CI* confidence interval, *CXR* chest x-ray, *IQR* interquartile range, *SD* standard deviation, *TTE* transthoracic echocardiography, *US* ultrasound

*Accuracy CXR investigated; TTE used as reference standard

^1^One-sided 97.5% confidence interval in case specificity is estimated to be 100%

^2^One-sided 97.5% confidence interval in case sensitivity is estimated to be 100%

^3^Exact confidence intervals (not taking into account between-study differences); GEE model not estimable as all controls were correctly identified

^4^Values shown as mean (SD) or median [IQR]

### Subgroup outcomes

**Fig. 5:**
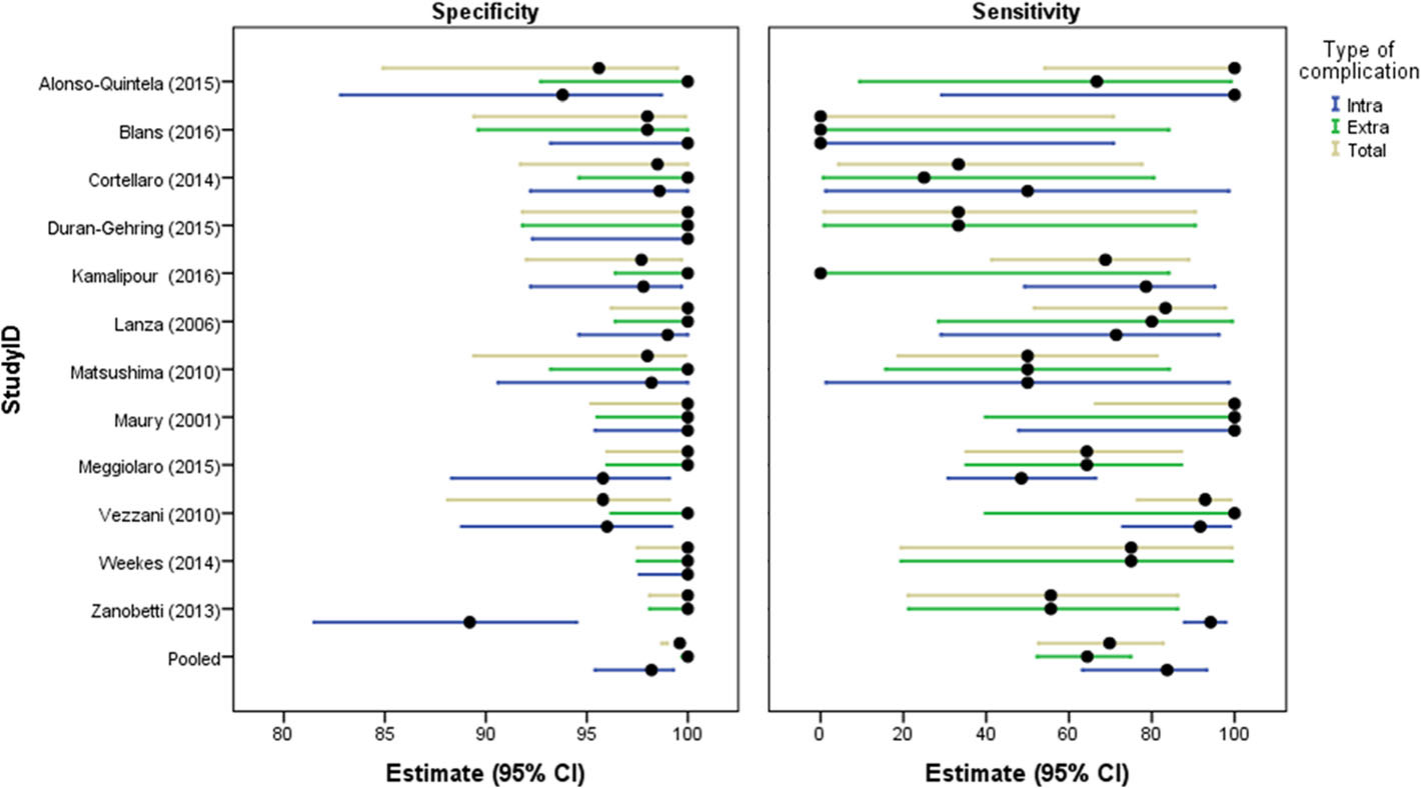
Forest plot for the specificity and sensitivity of ultrasound for detection of CVC-related complications distinguishing between intra- and extra-atrial malposition. The pooled specificity and sensitivity for intra- and extra-atrial malposition, and the specificity and sensitivity for each study individually. CI confidence interval

Pooled results from the subgroup analysis are shown in Table 2. The SCU group produced the highest specificity of 100% (95% CI: 94.4–100) but due to absent cases of malposition the sensitivity could not be calculated. The vascular US and TTE group yielded the highest sensitivity of 96.1% (95% CI: 79.7–99.4). The diagnostic accuracy of US to distinguish between intra- and extra-atrial malposition is shown in Table 3. Specificity of US for both intra- and extra-atrial malposition ranges from 95.6% (95% CI: 84.9–99.5) to 100% (95% CI: 98.1–100), whereas the sensitivity shows a distribution ranging from 0% (95% CI: 0–70.8) to 100% (95% CI:66.4–100), as shown in Fig. 5. A detailed description of the different US protocols with their reported respective advantages and disadvantages is given in Additional file 6. In all cases US was performed faster than CXR, with an average time of 2.83 min (95% CI: 2.77–2.89 min) for US compared to 34.7 min (95% CI: 32.6–36.7 min) and 46.3 min (95% CI: 44.4–48.2 min) for CXR performance and interpretation, respectively.

**Table 3:** Results of subgroup analysis

Study	Ultrasound protocol	Specificity^1^ (95% CI)	Sensitivity^2^ (95% CI)
All studies (pooled)			
Intra-atrial		97.4 (94.8–98.7)	73.5 (57.2–85.3)
Extra-atrial		100.0 (98.1–100.0)	55.6 (21.2–86.3)
Total		98.6 (97.2–99.3)	65.4 (50.7–77.6)
	TTE and CEUS		
Cortellaro [49]			
Intra-atrial		98.6 (92.2–100.0)	50.0 (1.2–98.7)
Extra-atrial		100.0 (94.6–100.0)	25.0 (0.6–80.6)
Total		98.5 (91.7–100.0)	33.3 (4.3–77.7)
Duran-Gehring [50]			
Intra-atrial		100.0 (92.3–100.0)	–^4^
Extra-atrial		100.0 (91.8–100.0)	33.3 (0.8–90.6)
Total		100.0 (91.8–100.0)	33.3 (0.8–90.6)
Kamalipour [52]			
Intra-atrial		97.8 (92.2–99.7)	78.6 (49.2–95.3)
Extra-atrial		100.0 (96.4–100.0)	0.0 (0.0–84.2)
Total		97.7 (92.0–99.7)	68.8 (41.3–89.0)
Lanza [53]			
Intra-atrial		99.0 (94.6–100.0)	71.4 (29.0–96.3)
Extra-atrial		100.0 (96.4–100.0)	80.0 (28.4–99.5)
Total		98.9 (94.3–100.0)	75.0 (42.8–94.5)
Weekes [55]			
Intra-atrial		100.0 (97.6–100.0)	–^4^
Extra-atrial		100.0 (97.5–100.0)	75.0 (19.4–99.4)
Total		100.0 (97.5–100.0)	75.0 (19.4–99.4)
	Vascular ultrasound and TTE		
Alonso-Quintela [58]			
Intra-atrial		94.0 (83.4–98.7)	100.0 (2.5–100.0)
Extra-atrial		100.0 (92.7–100.0)	100.0 (15.8–100.0)
Total		93.8 (82.8–98.7)	100.0 (29.2–100.0)
Maury [59]			
Intra-atrial		100.0 (95.4–100.0)	100.0 (47.8–100.0)
Extra-atrial		100.0 (95.5–100.0)	100.0 (39.8–100.0)
Total		100.0 (95.2–100.0)	100.0 (66.4–100.0)
	Vascular ultrasound, TTE and CEUS		
Blans [18]			
Intra-atrial		100.0 (93.2–100.0)	0.0 (0.0–70.8)
Extra-atrial		98.0 (89.6–100.0)	0.0 (0.0–84.2)
Total		98.0 (89.4–99.9)	0.0 (0.0–70.8)
Matsushima [19]			
Intra-atrial		98.2 (90.6–100.0)	50.0 (1.3–98.7)
Extra-atrial		100.0 (93.2–100.0)	50.0 (15.7–84.3)
Total		98.0 (89.4–99.9)	50.0 (18.7–81.3)
Meggiolaro^3^ [32]			
Intra-atrial		95.8 (88.3–99.1)	48.5 (30.8–66.5)
Extra-atrial		100.0 (96.0–100.0)	64.3 (35.1–87.2)
Total		100.0 (96.0–100.0)	64.3 (35.1–87.2)
Vezzani [31]			
Intra-atrial		96.0 (88.8–99.2)	91.7 (73.0–99.0)
Extra-atrial		100.0 (96.2–100.0)	100.0 (39.8–100.0)
Total		95.8 (88.1–99.1)	92.9 (76.5–99.1)
Zanobetti^3^ [61]			
Intra-atrial		89.2 (81.5–94.5)	94.2 (87.9–97.9)
Extra-atrial		100.0 (98.1–100.0)	55.6 (21.2–86.3)
Total		100.0 (98.1–100.0)	55.6 (21.2–86.3)

*CEUS* contrast enhance ultrasound, *TTE* transthoracic echocardiography

^1^One-sided 97.5% confidence interval (CI) in case specificity is estimated to be 100%

^2^One-sided 97.5% confidence interval in case sensitivity is estimated to be 100%

^3^Intra-atrial tip position was reported but was not considered to be a malposition

^4^No intra-atrial misplacements were detected

### Quality assessment

The risk of bias and applicability concerns of the included studies are summarized in Table 4. For a more detailed description see Additional file 5. No study scored low in all domains of the bias assessment. The risk of bias within the patient selection domain was considered low in 16 studies (64%). A higher risk assessment was mainly due to inappropriate exclusion and non-consecutive patient enrollment. The risk of bias in the index test domain was deemed low in 18 studies (72%). This risk of bias was most often scored high due to the lack of a threshold when using CEUS. Only four studies (16%) had a low risk of bias in the reference standard domain, primarily because studies used CXR to detect intra-atrial CVC misplacements. Within the domain of flow and timing, 18 studies (72%) scored low due to a variety of reasons. Only three studies (12%) had low applicability concerns regarding the reference standard. The remaining studies had inadequate numbers of operators. No concerns regarding applicability were found in either the patient selection or reference standard domains.

**Table 4:**
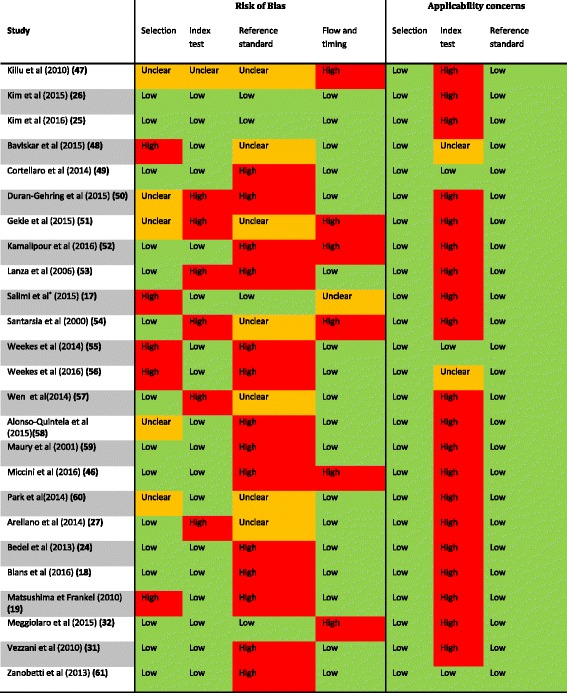
Quality assessment of included studies

*Accuracy CXR investigated; TTE used as reference standard

Orange is unclear risk of bias or applicability concern. Green is low risk of bias or applicability concern, and red is high risk of bias or applicability concern

## Discussion

The major findings of this systematic review and meta-analysis on the diagnostic accuracy of US to detect CVC malposition are a pooled specificity and sensitivity of 98.9 (95% CI: 97.8–99.5) and 68.2 (95% CI: 54.4–79.4), respectively. US was feasible in 96.8% of the cases. Furthermore, central line misplacement occurred in 6.8% and pneumothorax occurred in 1.1% of the population.

The prevalence of CVC malposition and pneumothorax in our systematic review and meta-analysis is in accordance with the published literature; the prevalence of primary CVC misplacement has been reported up to 6.7%, whereas pneumothorax normally ranges from 0.1–3.3% [3, 5, 6, 20, 21].

The limited sensitivity of US to detect CVC malposition can possibly be explained by the small a priori chance of developing post-procedural complications. Therefore, small changes in the number of false negatives could eventually cause dramatic changes in the sensitivity. This problem can persist even after pooling [22]. Another possible explanation for the low sensitivity might be an imperfect reference standard; some studies suggest that, in the absence of clinical symptoms, CXR should not be considered as a reliable diagnostic method [14]. There is a large inter-observer variability among radiologists in identifying the cavo-atrial junction on CXRs; therefore, reading of a bedside CXR alone may not be sufficiently accurate to identify intra-atrial tip position [6, 14, 15]. Off note, the risk of developing a serious complication, for example cardiac tamponade, secondary to CVC tip position in the right atrium is virtually zero [23]. Nevertheless, in spite of the low sensitivity, due to the high specificity and low prevalence the positive and negative predictive values are both excellent. Therefore, we can conclude that US is a suitable diagnostic modality to replace CXR.

Interestingly, the prevalence of CVC malposition may be reduced further by visualizing the guidewire during the insertion procedure [24–27]. In some studies echocardiography was performed during guidewire insertion in order to localize it as a hyper-echogenic line in the right atrium; subsequently, before CVC introduction the guidewire was slowly removed under US control until the “J” tip disappeared from the right atrium. If the guidewire was not visualized in the right atrium, a different view was attained and the wire was reinserted. Thus, this US protocol tends to reduce the occurrence of CVC malposition [24, 27]. The studies performed by Kim et al. incorporated a similar but slightly different per-procedural protocol (the SCU protocol as described in Additional file 6) [25, 26]. A major advantage of their protocol is that both CVC insertion and position control can be easily achieved by a single operator. Also, since the superior vena cava can readily be visualized via the right supraclavicular fossa, the advancement of the guidewire can be monitored fairly well during CVC insertion and any malposition is quickly recognized and corrected. The abovementioned protocols suggest that the rate of malposition only depends on the feasibility of US and could be as low as 0%.

CVC position, according to our meta-analysis, is best verified by vascular US combined with TTE. The SCU protocol could potentially be even better; since this protocol is performed during the insertion and procedure, misplacements rarely occur and, therefore, sensitivity could not be calculated. Theoretically, the best post-procedural protocol is an US method incorporating a scan of the jugular and subclavian vein bilaterally and visualizing the migration of the CVC tip into the heart through CEUS [28]. Surprisingly, in cases where CEUS was implemented in the study protocol an overall lower sensitivity was noted. This is probably due to the fact that studies incorporating CEUS generally deemed intra-atrial position of the catheter tip a misplacement, whereas in studies implementing only vascular US and TTE intra-atrial position was not always regarded as a malposition. Moreover, the vascular US and TTE group contained various pediatric studies where superior vena cava detection of the catheter tip is relatively easy [29, 30]. Finally, it has been debated whether the threshold of 2 s described by Vezzani and colleagues is an accurate indicator of correct CVC position [31]. More likely, the delay in appearance of microbubbles is dependent on the length of catheter used to inject the agitated saline. Subsequently, opacification of the right atrium only indicates an intravenous position of the CVC tip. Additionally, to assess CVC tip position, Meggiolaro et al. suggested that a cut-off value of 500 ms yields a better accuracy [32].

According to some ultrasound protocols two operators are required to insert the CVC and control its position at the same time with US. However, this is only necessary if either agitated saline is used to flush the line or a per-procedural protocol is being performed that visualizes the advancement of the guidewire. In any other case, one physician can insert the CVC and afterwards perform an ultrasonographic examination of the contralateral internal jugular vein, both subclavian veins, and the right atrium via the subcostal view. We suggest this information to be added to the protocol for US-guided CVC placement, recently published by Saugel and coworkers [33, 34]. We refer to Additional file 6 for a detailed description of all protocols and the number of operators needed.

Intra-atrial misplacement was more readily detected compared to extra-atrial misplacement. One possible explanation might be the fact that not all possible locations of extra-atrial malposition are detectable by US whereas the right atrium and ventricle are often easily scanned by TTE [35]. This would cause more false negatives to occur in the extra-atrial misplacement group and would therefore lead to a lower sensitivity.

Concerning the detection of pneumothorax, previous studies have already shown the advantages of US in comparison to CXR [36–38]. Furthermore, due to clear advantages, US has an increasing role in the critical care setting and ICU physicians are often trained in various US techniques [7, 39–42]. By combining the techniques of lung US and TTE we show that US could be a favorable method in detecting CVC-related complications in the ICU. To perform and interpret critical care US it is suggested that the majority of learning occurs during the first 20–30 practice studies and that many learners reached a plateau in their training [11, 43]. In general, bedside US has a good concordance with and multiple advantages over portable CXR, diminishing the role of CXR in the ICU [37, 38, 44].

Recently, the ability of US to detect malposition and pneumothorax following CVC insertion was investigated by another systematic review [45]. Its design contained some important differences compared to our meta-analysis. Firstly, we included far more CVC placements (2602 vs 1553) and studies (25 vs 15). Secondly, a strength of our study was that we included studies that used alternative reference standards; TEE, computed tomography, and intra-fluoroscopy were utilized in addition to CXR [26, 27, 46]. Thirdly, our study provides an accurate overview of the various US protocols used and their accuracy. Finally, we registered our study protocol at PROSPERO which is advised by the National Institute of Health Research (NIHR)*.* Besides study design, there were differences in results as well; we reported a similar pooled specificity of 98.9 (95% CI: 97.8–99.5) vs. 98 (95% CI: 97–99) but a considerably lower pooled sensitivity with a larger variation of 68.2 (95% CI: 54.4–79.4) vs. 82 (95% CI: 77–86). This discrepancy could be caused by the fact that we included more studies with smaller sample sizes, and studies without any positive cases.

Our meta-analysis has several limitations. Like all meta-analyses it is sensitive for publication bias. Deek’s test was not significant indicating no evidence of strong publication bias. Prevalence was low and seven studies did not have any occurrence of CVC-related complications. These studies did not provide information regarding the sensitivity. Moreover, the small number of positive cases reported in the included studies causes uncertainty and a large variation regarding the sensitivity estimates. In addition, specificity was found to be very high with no false positives in several studies. For these reasons we could not use a bivariate model as is often used in meta-analysis for diagnostic studies to jointly pool specificity and sensitivity estimates. Another limitation is the substantial amount of statistical heterogeneity concerning specificity and sensitivity; this limits the ability to interpret the pooled data. Possible explanations for this problem are small study populations, limitations in study designs, differences in US techniques, and differences in outcomes. Subgroup analyses were performed on the protocols and on the location of malposition (intra- or extra-atrial) to attenuate this problem. Another limitation is the overall high risk of bias in the reference test domain since CXR is often not reliable for detecting intra-atrial tip position.

Further research is required to establish the viability of US as a diagnostic tool. Regarding the sensitivity, this review shows a substantial amount of statistical heterogeneity, often caused by small study populations in addition to the low prevalence of complications. Due to the low prevalence it is nearly impossible to correctly power a study that investigates immediate post-procedural complications of central venous cannulation. To address this problem and assess the sensitivity correctly we suggest a larger study should be performed that uses operators of different level of experience, and the ‘SCU’ protocol or the ‘vascular US and TTE’ protocol as these showed the most promising results. Furthermore, future research should aim to investigate factors contributing to intravenous CVC malposition and pneumothorax. Identifying those factors could lead to a situation in which only cases with a high chance of complications are investigated by either US or CXR, thus reducing the required number of patients. Additionally, to detect aberrant CVC position the use of microbubbles should be re-evaluated since it is unclear whether CEUS itself produces more false negatives or that alternative factors contribute to a lower sensitivity.

## Conclusion

The major findings of this systematic review and meta-analysis on the diagnostic accuracy of US to detect CVC malposition are a pooled specificity and sensitivity of 98.9 (95% CI: 97.8–99.5) and 68.2 (95% CI: 54.4–79.4), respectively. Therefore, US is an accurate and feasible diagnostic modality to detect CVC malposition and iatrogenic pneumothorax. Advantages of US over CXR are that it is performed faster and does not subject patients to radiation. Vascular US combined with transthoracic echocardiography is advised. However, results need to be interpreted with caution since included studies were often underpowered and had methodological limitations. A large multicenter study investigating optimal US protocol, among others, is needed.

## Additional files

Additional file 1: PRISMA 2009 checklist. An overview of all sections as indicated by the PRISMA guidelines with their corresponding pages in the review. (DOC 63 kb)

Additional file 2: Search strategy. An overview of the various terms used to search the PubMed, EMBASE and Cochrane Library databases and the results from the initial search on 4 October 2016 and the secondary search on 9 January 2017. (DOCX 39 kb)

Additional file 3: Eligibility and exclusion criteria. An overview of the eligibility and exclusion criteria used in this review. (DOCX 13 kb)

Additional file 4: Patient characteristics. An overview of several characteristics of the included studies, namely gender, age, weight and/or BMI, CVC location, and type of catheter. (DOCX 26 kb)

Additional file 5: Qualitative assessment of bias. An overview of the domains defined by QUADAS-2 tool to assess the risk of bias and applicability concerns for each of the included articles. (DOCX 45 kb)

Additional file 6: Ultrasound protocols. An overview of the ultrasonographic techniques used in the various protocols. (DOCX 15 kb)

## Abbreviations

CEUS: Contrast-enhanced ultrasound
CI: Confidence interval
CVC: Central venous catheter
CXR: Chest x-ray
GEE: Generalized estimating equation
ICU: Intensive care unit
SCU: Supraclavicular ultrasound
TEE: Transesophageal echocardiography
TTE: Transthoracic echocardiography
US: Ultrasound
