# Supplementary material for: Bedside ultrasound to detect central venous catheter misplacement and associated iatrogenic complications: a systematic review and meta-analysis
Source: Crit Care. 2018 Mar 13;22:65. doi: 10.1186/s13054-018-1989-x (PMC5851097; doi:10.1186/s13054-018-1989-x)
Supplement: Supplementary file 2 — Search strategy. An overview of the various terms used to search the PubMed, EMBASE and Cochrane Library databases and the results from the initial search on 4 October 2016 and the secondary search on 9 January 2017. (DOCX 39 kb) [file 13054_2018_1989_MOESM2_ESM.docx]

**Additional file 2: Appendix B – Search strategy**

This appendix lists the various terms used to search the PubMed, Embase and Cochrane Library databases and the results from the initial search on October 4^th^ 2016 and the secondary search on January 9^th^ 2017.

**Database Search Terms**

**PubMed Search Terms**

1. cvc

"Catheterization, Central Venous"[Mesh] OR CVC*[tiab] OR central venous catheter*[tiab] OR central catheter*[tiab] OR central venous line*[tiab] OR central line*[tiab] OR ((Catheter*[tiab] OR cannul*[tiab]) AND (central venous[tiab] OR central vein[tiab] OR jugular[tiab] OR subclavian[tiab]))

1. ultrasound

"Ultrasonography"[Mesh] OR ultrasound*[tiab] OR sonograph*[tiab] OR echograph*[tiab] OR echocardiograph*[tiab] OR echotomograph*[tiab] OR ultrasonic[tiab] OR ultrasonograph*[tiab] OR Doppler*[tiab]

1. placement OR complications

placement*[tiab] OR position*[tiab] OR malposition*[tiab] OR localiz*[tiab] OR confirmation[tiab] OR “complications"[Subheading] OR “adverse effects"[Subheading] OR complication*[tiab] OR adverse event*[tiab] OR "Pneumothorax"[Mesh] OR pneumothorax[tiab]

## Embase Search Terms

1. cvc

'central venous catheterization'/exp OR CVC*:ab,ti OR ‘central venous catheter*’:ab,ti OR ‘central catheter*’:ab,ti OR ‘central venous line*’:ab,ti OR ‘central line*’:ab,ti OR ((Catheter*:ab,ti OR cannul*:ab,ti) AND (‘central venous’:ab,ti OR ‘central vein’:ab,ti OR jugular:ab,ti OR subclavian:ab,ti))

1. ultrasound

'echography'/exp OR ultrasound*:ab,ti OR sonograph*:ab,ti OR echograph*:ab,ti OR echotomograph*:ab,ti OR echocardiograph*:ab,ti OR ultrasonic:ab,ti OR ultrasonograph*:ab,ti OR Doppler*:ab,ti

1. placement OR complications

placement*:ab,ti OR position*:ab,ti OR malposition*:ab,ti OR localiz*:ab,ti OR confirmation:ab,ti OR complication*:ab,ti OR ‘adverse event*’:ab,ti OR 'pneumothorax'/exp

OR pneumothorax:ab,ti

## Cochrane Library Search Terms

1. cvc

CVC* OR “central venous catheter*” OR “central catheter*” OR “central venous line*” OR “central line*” OR ((Catheter* OR cannul*) AND (“central venous” OR “central vein” OR jugular OR subclavian))

1. ultrasound

ultrasound* OR sonograph* OR echograph* OR echotomograph* OR echocardiograph* OR ultrasonic OR ultrasonograph* OR Doppler*

1. placement OR complications

placement* OR position* OR malposition* OR localiz* OR confirmation OR complication* OR “adverse event*” OR pneumothorax

**Database Searches October 4^th^ 2016
PubMed Search History**

| Search | Query | Results |
| --- | --- | --- |
| #4 | #10 AND #11 AND #12 | 2413 |
| #3 | placement*[tiab] OR position*[tiab] OR malposition*[tiab] OR localiz*[tiab] OR confirmation[tiab] OR “complications"[Subheading] OR “adverse effects"[Subheading] OR complication*[tiab] OR adverse event*[tiab] OR "Pneumothorax"[Mesh] OR pneumothorax[tiab] | 4935988 |
| #2 | "Ultrasonography"[Mesh] OR ultrasound*[tiab] OR sonograph*[tiab] OR echograph*[tiab] OR echocardiograph*[tiab] OR echotomograph*[tiab] OR ultrasonic[tiab] OR ultrasonograph*[tiab] OR Doppler*[tiab] | 546389 |
| #1 | "Catheterization, Central Venous"[Mesh] OR CVC*[tiab] OR central venous catheter*[tiab] OR central catheter*[tiab] OR central venous line*[tiab] OR central line*[tiab] OR ((Catheter*[tiab] OR cannul*[tiab]) AND (central venous[tiab] OR central vein[tiab] OR jugular[tiab] OR subclavian[tiab])) | 30849 |

### Embase Search History

| No. | Query | Results |
| --- | --- | --- |
| #6 | **#4** NOT (**'conference abstract'**/it OR **'conference review'**/it OR **'editorial'**/it OR **'letter'**/it OR **'note'**/it) | **2128** |
| #5 | **#4** AND (**'conference abstract'**/it OR **'conference review'**/it OR **'editorial'**/it OR **'letter'**/it OR **'note'**/it) | **1287** |
| #4 | **#1** AND **#2** AND **#3** | **3415** |
| #3 | **placement***:ab,ti OR **position***:ab,ti OR **malposition***:ab,ti OR **localiz***:ab,ti OR **confirmation**:ab,ti OR **complication***:ab,ti OR **'adverse event*'**:ab,ti OR 'pneumothorax'/exp OR pneumothorax:ab,ti | **2345449** |
| #2 | **'echography'**/exp OR **ultrasound***:ab,ti OR **sonograph***:ab,ti OR **echograph***:ab,ti OR **echotomograph***:ab,ti OR **echocardiograph***:ab,ti OR **ultrasonic**:ab,ti OR **ultrasonograph***:ab,ti OR **doppler***:ab,ti | **872764** |
| #1 | **'central venous catheterization'**/exp OR **cvc***:ab,ti OR **'central venous catheter*'**:ab,ti OR **'central catheter*'**:ab,ti OR **'central venous line*'**:ab,ti OR **'central line*'**:ab,ti OR (**catheter***:ab,ti OR **cannul***:ab,ti AND (**'central venous'**:ab,ti OR **'central vein'**:ab,ti OR **jugular**:ab,ti OR **subclavian**:ab,ti)) | **39436** |

### Cochrane Library Search History

| No. | Query | Results |
| --- | --- | --- |
| #1 | CVC* or "central venous catheter*" or "central catheter*" or "central venous line*" or "central line*" or ((Catheter* or cannul*) and ("central venous" or "central vein" or jugular or subclavian)):ti,ab,kw (Word variations have been searched) | 2252 |
| #2 | ultrasound* or sonograph* or echograph* or echotomograph* or echocardiograph* or ultrasonic or ultrasonograph* or Doppler*:ti,ab,kw (Word variations have been searched) | 30653 |
| #3 | placement* or position* or malposition* or localiz* or confirmation or complication* or "adverse event*" or pneumothorax:ti,ab,kw (Word variations have been searched) | 143701 |
| #4 | #1 AND #2 And #3 | 233 |

**Database Searches January 9^th^ 2017**

**PubMed Search History**

| No. | Query | Results |
| --- | --- | --- |
| #4 | Search (#1 AND #2 AND #3) | 2565 |
| #3 | Search (placement*[tiab] OR position*[tiab] OR malposition*[tiab] OR localiz*[tiab] OR confirmation[tiab] OR “complications"[Subheading] OR “adverse effects"[Subheading] OR complication*[tiab] OR adverse event*[tiab] OR "Pneumothorax"[Mesh] OR pneumothorax[tiab]) | 4992947 |
| #2 | Search ("Ultrasonography"[Mesh] OR ultrasound*[tiab] OR sonograph*[tiab] OR echograph*[tiab] OR echocardiograph*[tiab] OR echotomograph*[tiab] OR ultrasonic[tiab] OR ultrasonograph*[tiab] OR Doppler*[tiab]) | 584224 |
| #1 | Search ("Catheterization, Central Venous"[Mesh] OR CVC*[tiab] OR central venous catheter*[tiab] OR central catheter*[tiab] OR central venous line*[tiab] OR central line*[tiab] OR ((Catheter*[tiab] OR cannul*[tiab]) AND (central venous[tiab] OR central vein[tiab] OR jugular[tiab] OR subclavian[tiab]))) | 31210 |

## Embase Search History

| No. | Query | Results |
| --- | --- | --- |
| #5 | **#4** NOT (**'conference abstract'**/it OR **'conference review'**/it OR **'editorial'**/it OR **'letter'**/it OR **'note'**/it) | **2186** |
| #4 | **#1** AND **#2** AND **#3** | **3533** |
| #3 | **placement***:ab,ti OR **position***:ab,ti OR **malposition***:ab,ti OR **localiz*:**ab,ti OR **confirmation**:ab,ti OR **complication***:ab,ti OR **'adverse event*'**:ab,ti OR **'pneumothorax'**/exp OR **'pneumothorax'** OR **pneumothorax**:ab,ti | **2395259** |
| #2 | **'echography'**/exp OR **'echography'** OR **ultrasound***:ab,ti OR **sonograph***:ab,ti OR **echograph***:ab,ti OR **echotomograph***:ab,ti OR **echocardiograph***:ab,ti OR **ultrasonic**:ab,ti OR **ultrasonograph***:ab,ti OR **doppler***:ab,ti | **898872** |
| #1 | **'central venous catheterization'**/exp OR **'central venous catheterization'** OR **cvc***:ab,ti OR **'central venous catheter*'**:ab,ti OR **'central catheter*'**:ab,ti OR **'central venous line*'**:ab,ti OR **'central line*'**:ab,ti OR (**catheter***:ab,ti OR **cannul***:ab,ti AND (**'central venous'**:ab,ti OR **'central vein'**:ab,ti OR **jugular**:ab,ti OR **subclavian**:ab,ti)) | **40297** |

### Cochrane Library Search History

| No. | Query | Results |
| --- | --- | --- |
| #1 | CVC* or "central venous catheter*" or "central catheter*" or "central venous line*" or "central line*" or ((Catheter* or cannul*) and ("central venous" or "central vein" or jugular or subclavian)):ti,ab,kw (Word variations have been searched) | 2549 |
| #2 | ultrasound* or sonograph* or echograph* or echotomograph* or echocardiograph* or ultrasonic or ultrasonograph* or Doppler*:ti,ab,kw (Word variations have been searched) | 34881 |
| #3 | placement* or position* or malposition* or localiz* or confirmation or complication* or "adverse event*" or pneumothorax:ti,ab,kw (Word variations have been searched) | 196677 |
| #4 | #1 AND #2 And #3 | 341 |
