# Supplementary material for: Bedside ultrasound to detect central venous catheter misplacement and associated iatrogenic complications: a systematic review and meta-analysis
Source: Crit Care. 2018 Mar 13;22:65. doi: 10.1186/s13054-018-1989-x (PMC5851097; doi:10.1186/s13054-018-1989-x)
Supplement: Supplementary file 3 — Eligibility and exclusion criteria. An overview of the eligibility and exclusion criteria used in this review. (DOCX 13 kb) [file 13054_2018_1989_MOESM3_ESM.docx]

**Additional file 3: Appendix C - Eligibility and exclusion criteria**

Studies included in this review were required to have human subjects with a central venous catheter (CVC) in the subclavian or internal jugular vein. The index test should consist of ultrasound techniques to confirm correct placement of CVCs and to detect potential complications. The reference standard should be able to correctly confirm CVC placement and its potential complications.

Studies were excluded if they were reviews/comments/editorials/correspondences or if they had a case report/series design; if the index test consisted of TEE, or US for any other reason than the confirmation of CVC placement or detection of mechanical complications; if no reference standard was used; if it was impossible to determine accuracy of the index test; if no English/Spanish/Portuguese version of the article was available.
