# Supplementary material for: Bedside ultrasound to detect central venous catheter misplacement and associated iatrogenic complications: a systematic review and meta-analysis
Source: Crit Care. 2018 Mar 13;22:65. doi: 10.1186/s13054-018-1989-x (PMC5851097; doi:10.1186/s13054-018-1989-x)
Supplement: Supplementary file 4 — Patient characteristics. An overview of several characteristics of the included studies, namely gender, age, weight and/or BMI, CVC location, and type of catheter. (DOCX 26 kb) [file 13054_2018_1989_MOESM4_ESM.docx]

**Additional file 4: Appendix D – Patient characteristics**

This appendix describes several characteristics of the included studies, namely gender, age and weight and/or BMI. Furthermore, the locations and of inserted catheters and CVC type are also listed.

| Study (year) | **M/F** | **Age in years (SD) [IQR]** | **Weight in kg/BMI (SD) [IQR]** | **IJV** | **SV** | **PICC** | **FV** | **CVC type** |
| --- | --- | --- | --- | --- | --- | --- | --- | --- |
| Alonso-Quintela et al. (2015) | - | 6ms, [2-28] | 6.5 [4.8-20]/- | 17 | 9 | 2 | 23 | Not reported |
| Arellano et al (2014) | 72/28 | 64 [42-86] | -/29 [18-47] | 100 | 0 | 0 | 0 | Not reported |
| Baviskar et al. (2015) | 15/10 | 9-83 | -/- | 20 | 2 | 3 | 0 | Not reported |
| Bedel et al (2013) | 66/32 | 56 (17) | -/24 (3) | 47 | 54 | 0 | 0 | Quad lumen |
| Blans et al. (2016) | 25/28 | 64 (12.8) | -/26.7 (5.2) | 50 | 3 | 0 | 0 | Tripe lumen |
| Cortellaro et al. (2014) | 44/27 | 71 (10) | -/- | 61 | 10 | 0 | 0 | Triple lumen |
| Duran-Gehring et al. (2015) | 18/32 | 58 (15.3) | -/- | 42 | 4 | 0 | 0 | Triple lumen |
| Gekle et al. (2015) | - | - | -/- | 68 | 0 | 0 | 0 | Not reported |
| Kamalipour (2016) et al. | 62/42 | 58.2 (12) | 67.7 (15)/ 25.2 (13) | 93 | 11 | 0 | 0 | Triple lumen |
| Killu et al. (2010) | - | - | - | 5 | 0 | 0 | 0 | Not reported |
| Kim et al. (2014) | 9/9 | 64 (14) | 86.7 (16.5) /29 (4) | 18 | 0 | 0 | 0 | Dual or quad lumen |
| Kim et al. (2016) | 30/18 | 66.5 (1.8) | -/28.72 (0.9) | 48 | 0 | 0 | 0 | Dual or triple lumen |
| Lanza et al. (2006) | 75/32 | 31.7ds [1ds-7] | - | 63 | 44 | 0 | 0 | Single lumen |
| Matsushima et Frankel (2010) | 36/23 | 51.4 (15.6) | - | 13 | 29 | 41 | 0 | Triple lumen |
| Maury et al. (2001) | 51/30 | 63.8 (16) | - | 15 | 70 | 0 | 0 | Dual lumen |
| Meggiolaro et al. (2015) | 70/35 | 74 (6.5) | 70 (5)/25 (3) | 79 | 26 | 0 | 0 | Dual lumen |
| Miccini et al. (2016) | 176/ 126 | 67.3 (3.5) | - | 302 | 0 | 0 | 0 | Not reported |
| Park et al. (2014) | 49/57 | 4.1ms (3.1) | 5.9 (2.0)/- | 106 | 0 | 0 | 0 | Single lumen |
| Salimi et al. (2015) | 54/28 | 57.37 (18.91) | -/23.64 (4.55) | 82 | 0 | 0 | 0 | Not reported |
| Santarsia et al. (2000) | - | - | - | 54 | 0 | 0 | 0 | Dual lumen |
| Vezzani et al. (2010) | 70/41 | 60 (18) | -/26 (5) | 26 | 85 | 0 | 0 | Dual lumen |
| Weekes et al. (2014) | 73/83 | 62.1 (15.6) | - | 137 | 14 | 0 | 0 | Triple lumen |
| Weekes et al. (2016) | 68/80 | 58.4 (15.7) | - | 107 | 28 | 0 | 15 | Triple lumen |
| Wen et al. (2014) | 99/103 | 57 (17-97) | - | 219 | 0 | 0 | 0 | Dual lumen |
| Zanobetti et al. (2014) | 101/ 109 | 72 (27) | - | 124 | 80 | 0 | 0 | Triple lumen |

BMI = Body Mass Index, ds = days, F = Female, FV = Femoral Vein, IJV = Internal Jugular Vein, IQR = Interquartile Range, kg = kilogram, M = Male, ms = months, PICC = Peripherally Inserted Catheter, SD = Standard Deviation, SV = Subclavian Vein
