# Supplementary material for: Bedside ultrasound to detect central venous catheter misplacement and associated iatrogenic complications: a systematic review and meta-analysis
Source: Crit Care. 2018 Mar 13;22:65. doi: 10.1186/s13054-018-1989-x (PMC5851097; doi:10.1186/s13054-018-1989-x)
Supplement: Supplementary file 5 — Qualitative assessment of bias. An overview of the domains defined by QUADAS-2 tool to assess the risk of bias and applicability concerns for each of the included articles. (DOCX 45 kb) [file 13054_2018_1989_MOESM5_ESM.docx]

**Additional file 5: Appendix E – Qualitative assessment of bias**

To assess the risk of bias and applicability, four domains were evaluated concerning patient selection, index test, reference standard, and flow and timing. Risk of bias concerning patient selection was low when patients were consecutively or randomly enrolled, a case control design was avoided, and the study did not have any inappropriate exclusions. Risk of bias concerning index test was regarded low when index test results were interpreted without knowledge of the results of the reference standard. Risk of bias concerning reference standard was considered to be low if the reference standard was likely to correctly classify the target condition and the results were interpreted without knowledge of the results of the index test. The risk of bias with regard to flow and timing was deemed low, if there was an appropriate interval between index test and reference standard, if all patients received a reference standard, whether all patients received the same reference standard, and if all patients were included in the analysis. The figures below show the QUADAS-2 tool used to assess risk of bias an applicability. An individual assessment of every included study is described subsequently.

**DOMAIN 1: PATIENT SELECTION**

**A. Risk of Bias**

*Describe methods of patient selection:*

Was a consecutive or random sample of patients enrolled? Yes/No/Unclear

Was a case-control design avoided? Yes/No/Unclear

Did the study avoid inappropriate exclusions? Yes/No/Unclear

**Could the selection of patients have introduced bias?** **RISK: LOW/HIGH/UNCLEAR**

**B. Concerns regarding applicability**

*Describe included patients (prior testing, presentation, intended use of index test and setting)****:***

**Is there concern that the included patients do not match CONCERN: LOW/HIGH/UNCLEAR the review question?**

**DOMAIN 2: INDEX TEST(S)**

**If more than one index test was used, please complete for each test.**

**A. Risk of Bias**

*Describe the index test and how it was conducted and interpreted:*

Were the index test results interpreted without

knowledge of the results of the reference standard? Yes/No/Unclear

If a threshold was used, was it pre-specified? Yes/No/Unclear

**Could the conduct or interpretation of the index test** **RISK: LOW /HIGH/UNCLEAR**

**have introduced bias?**

**B. Concerns regarding applicability**

**Is there concern that the index test, its conduct, or** **CONCERN: LOW /HIGH/UNCLEAR**

**interpretation differ from the review question**

**DOMAIN 3: REFERENCE STANDARD**

**A. Risk of Bias**

*Describe the reference standard and how it was conducted and interpreted:*

Is the reference standard likely to correctly classify

the target condition? Yes/No/Unclear

Were the reference standard results interpreted without

knowledge of the results of the index test? Yes/No/Unclear

**Could the reference standard, its conduct, or its** **RISK: LOW /HIGH/UNCLEAR**

**interpretation have introduced bias?**

**B. Concerns regarding applicability**

**Is there concern that the target condition as defined by CONCERN: LOW /HIGH/UNCLEAR**

**the reference standard does not match the review**

**question?**

**DOMAIN 4: FLOW AND TIMING**

**A. Risk of Bias**

Describe any patients who did not receive the index test(s) and/or reference standard or who were excluded from the 2x2 table (refer to flow diagram):

Describe the time interval and any interventions between index test(s) and reference standard:

Was there an appropriate interval between index test(s)

and reference standard? Yes/No/Unclear

Did all patients receive a reference standard? Yes/No/Unclear

Did patients receive the same reference standard? Yes/No/Unclear

Were all patients included in the analysis? Yes/No/Unclear

**Could the patient flow have introduced bias? RISK: LOW /HIGH/UNCLEAR**

**Alonso-Quintela et al. (2015)**

*Selection:* The risk of bias is unclear because it is not distinctly stated how patients are enrolled and whether there were inappropriate exclusions. Concerns regarding applicability are low.

*Index test:* The risk of bias is low because operators were blinded to CXR results. A threshold was used for CEUS. Concerns regarding applicability are high since one operator performed US examinations.

*Reference standard:* The risk of bias is high because CXR was used to detect intra-atrial CVC misplacements. Concerns regarding applicability are low.

*Flow and timing:* The risk of bias is low because CXR was performed after US examinations.

**Arellano et al. (2014)**

*Selection:* The risk of bias is low because patients were enrolled consecutively. Concerns regarding applicability are low since enrolled patients match the review question.

*Index test:* The risk of bias is high because no threshold was used for CEUS. It is unclear whether operators were blinded to CXR results. Concerns regarding applicability are high since CVCs in SCV were excluded.

*Reference standard:* The risk of bias is unclear because it is uncertain if operators were blinded to US results.

*Flow and timing:* The risk of bias is low because CXR was performed after US examinations.

**Baviskar et al. (2015)**

*Selection:* The risk of bias is high because patients were inappropriately excluded. Concerns regarding applicability are low because enrolled patients match the review question.

*Index test:* The risk of bias is low because operators were blinded to CXR results. A threshold was used for CEUS. Concerns regarding applicability are unclear since it was not distinctly stated how many operators performed US examinations.

*Reference standard:* The risk of bias is unclear because it is uncertain if operators were blinded to US results. Concerns regarding applicability are low.

*Flow and timing:* The risk of bias is low because CXR was performed after US examinations.

**Bedel et al. (2013)**

*Selection:* The risk of bias is low because patients were enrolled consecutively. Concerns regarding applicability are low since enrolled patients match the review question.

*Index test:* The risk of bias is low because operators were blinded to CXR results. Concerns regarding applicability are high since a single operator performed US examinations.

*Reference test:* The risk of bias is high because CXR was used to detect intra-atrial misplacements. Concerns regarding applicability are low.

*Flow and timing:* The risk of bias is low because CXR was performed after US examinations.

**Blans et al. (2016)**

*Selection:* The risk of bias is low because patients were enrolled consecutively. Concerns regarding applicability are low since enrolled patients match the review question.

*Index test:* The risk of bias is low because operators were blinded to CXR results and a threshold was used for CEUS. Concerns regarding applicability are high since two operators performed US examinations.

*Reference standard:* The risk of bias is high because CXR was used to detect intra-atrial CVC misplacements. Concerns regarding applicability are low.

*Flow and timing:* The risk of bias is low because CXR was performed after US examinations.

**Cortellaro et al. (2014)**

*Selection:* The risk of bias is low because patients were enrolled consecutively. Concerns regarding applicability are low since enrolled patients match the review question.

*Index test:* The risk of bias is low because operators were blinded to CXR results and a threshold was used for CEUS. Concerns regarding applicability are low.

*Reference standard:* The risk of bias is high because CXR was used to detect intra-atrial CVC misplacements. Concerns regarding applicability are low.

*Flow and timing:* The risk of bias is low because CXR was performed after US examinations.

**Duran-Gehring et al. (2015)**

*Selection:* The risk of bias is unclear because it not clearly stated if there were inappropriate exclusions. Concerns regarding applicability are low since patients match the review question.

*Index test:* The risk of bias is high because no threshold was used for CEUS. Concerns regarding applicability are high since two operators performed US examinations.

*Reference standard:* The risk of bias is high because CXR was used to detect intra-atrial CVC misplacements. Concerns regarding applicability are low.

*Flow and timing:* The risk of bias is low because CXR was performed after US examinations.

**Gekle et al. (2015)**

*Selection:* The risk of bias is unclear because it not distinctly stated whether patients were enrolled consecutively or randomly. It is unclear if there were inappropriate exclusions. Concerns regarding applicability are low since patients match the review question.

*Index test:* The risk of bias is high because no threshold was used for CEUS. Concerns regarding applicability are high since CVCs in SCV were excluded and the amount of operators in unclear.

*Reference standard:* The risk of bias is unclear because it is uncertain whether the operators were blinded to US results. Concerns regarding applicability are low.

*Flow and timing:* The risk of bias is high because patients were excluded from analysis.

**Kamalipour et al. (2015)**

*Selection:* The risk of bias is low because patients were enrolled consecutively. Concerns regarding applicability are low since enrolled patients match the review question.

*Index test:* The risk of bias is low because operators were blinded to CXR results and a threshold was used for CEUS. Concerns regarding applicability are high since a single operator performed US examinations.

*Reference standard:* The risk of bias is high because CXR was used to detect intra-atrial CVC misplacements. Concerns regarding applicability are low.

*Flow and timing:* The risk of bias is high because cardiac surgery was performed between US and CXR. Not every patient was included in analysis.

**Killu et al. (2010)**

*Selection:* The risk of bias is unclear because it is not distinctly stated how patients were enrolled or whether a case-control design was avoided. Concerns regarding applicability are low.

*Index test:* The risk of bias is unclear because it is uncertain if operators were blinded to CXR results. Concerns regarding applicability are high since CVCs in SCV were excluded. One operator performed US examinations.

*Reference test:* The risk of bias is unclear because it is uncertain if operators were blinded to US results. Concerns regarding applicability are low.

*Flow and timing:* The risk of bias is high because CXR was performed before US examinations.

**Kim et al. (2016)**

*Selection:* The risk of bias is low because patients were enrolled consecutively. Concerns regarding applicability are low since enrolled patients match the review question.

*Index test:* The risk of bias is low because operators were blinded to CXR results. Concerns for applicability are high since CVCs in IJV were excluded and one operator performed US examinations.

*Reference standard:* The risk of bias is low because operators were blinded to US results. Concerns regarding applicability are low.

*Flow and timing:* The risk of bias is low because CXR was performed after US examinations.

**Kim et al. (2015)**

*Selection:* The risk of bias is low because patients were enrolled consecutively. Concerns regarding applicability are low since enrolled patients match the review question.

*Index test:* The risk of bias is low because operators were blinded to CXR results. Concerns for applicability are high since CVCs in SCV were excluded and two operators performed US examinations.

*Reference standard:* The risk of bias is low because operators were blinded to US results. Concerns regarding applicability are low.

*Flow and timing:* The risk of bias is low because CXR was performed after US examinations.

**Lanza et al. (2006)**

*Selection:* The risk of bias is low because patients were enrolled consecutively. Concerns regarding applicability are low since enrolled patients match the review question.

*Index test:* The risk of bias is high because no threshold was used for CEUS. Concerns regarding applicability are high since one operator performed US examinations.

*Reference standard:* The risk of bias is high because CXR was used to detect intra-atrial CVC misplacements. Concerns regarding applicability are low.

*Flow and timing:* The risk of bias is low because CXR was performed after US examinations.

**Matsushima & Frankel (2010)**

*Selection:* The risk of bias is high because inappropriate exclusions were not avoided. Concerns regarding applicability are low since patients match the review question.

*Index test:* The risk of bias is low because operators were blinded to CXR results. Concerns regarding applicability are high since one operator performed US examinations.

*Reference standard:* The risk of bias is high because CXR was used to detect intra-atrial CVC misplacements. Concerns regarding applicability are low.

*Flow and timing:* The risk of bias is low because CXR was performed after US examinations.

**Maury et al. (2001)**

*Selection:* The risk of bias is low because patients were enrolled consecutively. Concerns regarding applicability are low since enrolled patients match the review question.

*Index test:* The risk of bias is low because operators were blinded to CXR results. Concerns regarding applicability are high since three inexperienced operators performed US examinations.

*Reference standard:* The risk of bias is high because CXR is used to detect intra-atrial CVC misplacements. It is unclear if the operators were blinded to the US results. Concerns regarding applicability are low.

*Flow and timing:* The risk of bias is low because CXR was performed after US examinations.

**Meggiolaro et al. (2015)**

*Selection:* The risk of bias is low because patients were enrolled consecutively. Concerns regarding applicability are low since enrolled patients match the review question.

*Index test:* The risk of bias is low because operators were blinded to CXR results. Concerns regarding applicability are high since a single operator performed all US examinations.

*Reference standard:* The risk of bias is low because operators were blinded to US results. Concerns regarding applicability are low.

*Flow and timing:* The risk of bias is high because patients received CXR before and after US examinations.

**Miccini et al. (2016)**

*Selection:* The risk of bias is low because patients were enrolled consecutively. Concerns regarding applicability are low since enrolled patients match the review question.

*Index test:* The risk of bias is low because operators were blinded to IF results. Concerns regarding applicability are high because CVCs in SCV were excluded and two operators performed all US examinations.

*Reference standard:* The risk of bias is high because CVC was corrected before IF performance. Concerns regarding applicability are low.

*Flow and timing:* The risk of bias is high because not every patient received the reference standard.

**Park et al. (2014)**

*Selection:* The risk of bias is unclear because it is not stated how patients were enrolled. It is unclear if inappropriate exclusion criteria were used. Concerns regarding applicability are low.

*Index test:* The risk of bias is low because operators were blinded to CXR results. Concerns regarding applicability are high because CVCs in SCV were excluded.

*Reference standard:* The risk of bias is unclear because it is no stated whether the CXR operator were blinded to US results. Concerns regarding applicability are low.

*Flow and timing:* The risk for bias is low because CXR was performed after US examinations.

**Salimi et al. (2015)**

*Selection:* The risk of bias is high because patients were not enrolled consecutively or randomly. Inappropriate exclusions were not avoided. Concerns regarding applicability are low since enrolled patients match the review question.

*Index test:* The risk of bias is low because operators were blinded to CXR results. Concerns regarding applicability are high since one operator performed all ultrasound examinations and CVCs in SCV were excluded.

*Reference standard:* The risk of bias is low because operators were blinded to US results. Concerns regarding applicability are low.

*Flow and timing:* The risk for bias is unclear because uncertain whether all patients were included in analysis. The time-interval between US and CXR is also unclear.

**Santarsia et al. (2000)**

*Selection:* The risk of bias is low because patients were enrolled consecutively. Concerns regarding applicability are low since enrolled patients match the review question.

*Index test:* The risk of bias is high because no threshold was used for CEUS. It is unclear how many operators performed CEUS. Concerns regarding applicability are high since CVCs in the SCV were excluded.

*Reference standard:* The risk of bias is unclear because it is not stated whether CXR operators were blinded to CEUS results. Concerns regarding applicability are low.

*Flow and timing:* The risk of bias is high because some patients did not receive CXR.

**Vezzani et al. (2010)**

*Selection:* The risk of bias is low because patients were enrolled consecutively. Concerns regarding applicability are low since enrolled patients match the review question.

*Index test:* The risk of bias is low since operators were blinded to CXR results and a threshold was used for CEUS. Concerns regarding applicability are high since a single operator performed CEUS.

*Reference standard:* Risk of bias is high because CXR was used to detect intra-atrial CVC misplacements. It is unclear whether CXR operators were blinded to the results of CEUS. Concerns regarding applicability are low.

*Flow and timing:* The risk of bias is low because CXR was performed after US examinations.

**Weekes et al. (2014)**

*Selection:* The risk of bias is high because patients were not enrolled consecutively. Concerns regarding applicability are low since enrolled patients match the review question.

*Index test:* The risk of bias is low since operators were blinded to CXR results and a threshold was used for CUES. Concerns regarding applicability are low.

*Reference standard:* Risk of bias is high since CXR was used to detect intra-atrial CVC misplacements. Concerns regarding applicability are low.

*Flow and timing:* The risk of bias is low because CXR was performed after US examinations.

**Weekes et al. (2016)**

*Selection:* The risk of bias is high because patients were not enrolled consecutively. Concerns regarding applicability are low since enrolled patients match the review question.

*Index test:* The risk of bias is low since operators were blinded to CXR results and a threshold was used for CEUS. Concerns regarding applicability are unclear since there were an unspecified amount of investigators.

*Reference standard:* Risk of bias is high since CXR was used to detect intra-atrial CVC misplacements. Concerns regarding applicability are low.

*Flow and timing:* The risk of bias is low because CXR was performed after US examinations.

**Wen et al. (2014)**

*Selection:* The risk of bias is low because patients were enrolled consecutively. Concerns regarding applicability are low since enrolled patients match the review question.

*Index test:* The risk for bias is high because no threshold was used for performing CEUS. It was unclear whether the CEUS operators were blinded to the results of CXR. Concerns regarding applicability are high since CVCs in the SCV were excluded.

*Reference standard:* Risk of bias is unclear because it is not stated if CXR operators were blinded to CEUS results. Concerns regarding applicability are low.

*Flow and timing:* The risk of bias is low because CXR was performed after US examinations.

**Zanobetti et al. (2013)**

*Selection:* The risk of bias is low because patients were enrolled consecutively. Concerns regarding applicability are low since enrolled patients match the review question.

*Index test:* The risk of bias is low because ultrasound operators were blinded to CXR results. Concerns regarding applicability are low.

*Reference standard:* The risk of bias is high because CXR was used to detect intra-atrial CVC misplacements. Concerns regarding applicability are low.

*Flow and timing:* The risk of bias is low because CXR was performed after US examinations.
