# Supplementary material for: Bedside ultrasound to detect central venous catheter misplacement and associated iatrogenic complications: a systematic review and meta-analysis
Source: Crit Care. 2018 Mar 13;22:65. doi: 10.1186/s13054-018-1989-x (PMC5851097; doi:10.1186/s13054-018-1989-x)
Supplement: Supplementary file 6 — Ultrasound protocols. An overview of the ultrasonographic techniques used in the various protocols. (DOCX 15 kb) [file 13054_2018_1989_MOESM6_ESM.docx]

**Additional file 6: Appendix F**

**Ultrasound protocols**

**Supraclavicular ultrasound**

This method of detecting CVC position was utilized by three studies: Kim et al. (2014), Kim et al. (2016) and Killu et al. (2010). These studies used a micro convex ultrasound transducer by placing it in the right supraclavicular fossa. After an appropriate view of the SVC to the right pulmonary artery (RPA) was obtained, the guidewire was inserted under real-time US guidance. The CVC was advanced along the guidewire, after which its position was confirmed by the US.
A major advantage of this technique is that both CVC insertion and position control can be easily completed by a single operator. Also, since the SVC can readily be visualized via the right supraclavicular fossa, the advancement of the guidewire can be monitored fairly well during CVC insertion and any malposition is quickly recognized. Intra-cardiac tip malposition, however, cannot be investigated; due to its material properties, the CVC tip in contrast to the guidewire itself cannot be visualized. Another limitation of this method is that to achieve the optimal ultrasound quality a micro convex transducer should be used, which is designed for pediatric ultrasound; hence, in institutions without pediatric care its availability might be impaired.

**TTE and CEUS**

A total of eleven studies used this protocol for CVC detection: Baviskar et al. (2015), Cortellaro et al. (2014), Duran-Gehring et al. (2015), Gekle et al. (2015), Kamalipour et al. (2016), Lanza et al. (2006), Salimi et al. (2015), Santarsia et al. (2000), Weekes et al. (2014), Weekes et al. (2016) and Wen et al. (2014). After CVC placement, with or without US guidance, the RA was visualized using an apical four-chamber view or a subcostal view. Once a clear image was acquired an assistant flushed the distal CVC port with either 10- or 20mL saline solution (NaCl 0.9%) and 1mL air. By vigorously shaking the syringe microbubbles were created, which were injected after the remaining air was removed from the syringe. If the CVC was positioned correctly, the microbubbles were visible on the TTE within two seconds. A longer delay indicated aberrant placement.
This protocol can be performed quickly and post-procedural; besides, in combination with lung-ultrasound the most important iatrogenic complications can be detected. However, the CVC tip is only indirectly visible through the saline-flush’s microbubbles. US itself cannot detect the CVC tip and therefore cannot specify the position of a misplaced catheter. Two operators are required to perform this protocol

**Vascular ultrasound and TTE**

This US protocol was used by six studies: Alonso-Quintela et al. (2015), Maury et al. (2001), Miccini et al. (2016), Park et al. (2014), Arellano et al. (2014) and Bedel et al. (2013). Following CVC insertion, the RA and SVC were observed, mainly through the subcostal view. If the catheter tip could not be visualized through this method, the IJVs and SVs were scanned in search for a malposition of the CVC. An advantage of this method is that possible CVC tip malpositions in both the IJV or SV, and the RA can be detected. A limitation is that it is very hard to get a clear image in obese patients or patients who recently underwent cardiac surgery. One operator is required to perform this protocol

**Vascular ultrasound, TTE and CEUS**

Five studies employed this method: Blans et al. (2016), Matsushima et Frankel (2010), Meggiolaro et al. (2015), Vezzani et al. (2010) and Zanobetti et al. (2013). This protocol combined the techniques from the previous two methods. After CVC insertion, the IJV and SV were scanned to look for aberrant positions. Following this, the RA and SVC were visualized and a thoroughly shaken 10- or 20mL saline solution (NaCl 0.9%) was injected after excess air was removed. Again, a delay of over two seconds indicated an extra-atrial malposition. Direct visualization of the microbubbles suggested an intra-atrial malposition.
(Dis)advantages matched those mentioned in the previous two sections. Two operators are required to perform this protocol.

**Pneumothorax**

Eleven studies used ultrasonography to check for pneumothoraxes: Duran-Gehring et al. (2015), Gekle et al. (2015), Lanza et al. (2006), Maury et al. (2001), Miccine et al. (2016), Bedel et al. (2013), Blans et al. (2016), Matsushima et Frankel (2010), Meggiolaro et al. (2015), Vezzani et al. (2010) and Zanobetti et al. (2013). Pneumothorax was detected using lung-ultrasound and defined by an absence of the gliding sign and vertical artifacts (B-lines), and the presence of a lung point.
